# Food Insecurity: Is Leagility a Potential Remedy?

**DOI:** 10.3390/foods12163138

**Published:** 2023-08-21

**Authors:** Aleksandra Kowalska, Sophia Lingham, Damian Maye, Louise Manning

**Affiliations:** 1 Institute of Economics and Finance, Maria Curie-Skłodowska University, pl. Marii Curie-Skłodowskiej 5, 20-031 Lublin, Poland; aleksandra.kowalska@mail.umcs.pl; 2 School of Agriculture, Food and Environment, Royal Agricultural University, Stroud Road, Cirencester GL7 6JS, UK; sophia.lingham@student.rau.ac.uk; 3 Countryside and Community Research Institute, University of Gloucestershire, Swindon Road, Cheltenham GL50 4AZ, UK; dmaye@glos.ac.uk; 4 Lincoln Institute for Agri Food Technology, University of Lincoln, Riseholme Park, Lincoln LN2 2LG, UK

**Keywords:** lean, agile, leagility, food system, operationalisation, remedies, food insecurity

## Abstract

In the wake of the COVID-19 pandemic, and Ukraine–Russian conflict, both significant geo-political and socio-economic shocks to the global food system and food insecurity has risen across the world. One potential remedy to reduce the level of food insecurity is to move from a lean just-in-time food system to one where there is more resilience through greater agility both in routine supply operations and also in the event of an emergency situation. The aim of this critical perspectives paper was to firstly reflect on the concepts of lean, agility, and ‘leagility’. Then, this study considered the ability of individual organisations and the whole food system to be resilient, adaptive, enable the elimination of waste, reduce inefficiency, and assure the consistent delivery to market requirements in terms of both volume, safety, and quality. Promoting the concept of leagility together with advocating resilient, sustainable practices that embed buffer and adaptive capacity, this paper positions that increasing digitalisation and improving business continuity planning can ensure effective operationalisation of supply chains under both normal and crisis situations, ultimately reducing the risk of food insecurity at personal, household, and community levels.

## 1. Introduction

The term ‘supply chain’ suggests a linear chain from suppliers to customers, where one organisation supplies resources and materials to another [1]. Some reject this oversimplified linear conception of these sequential dyadic relationships and describe a supply chain as *“a network via a set of ‘nodes’ that represent autonomous business units as firms who are able to exercise sovereign choices, and a set of ‘connections’ that link these firms together for the purposes of creating products or services”* [2] (p. 444). The critical connections are the presence of contracts, material flows, financial flows, and information flows. These ‘flows’ are more specifically described as vertical, horizontal, and bidirectional exchange relationships between organisations in a network model, with highly complex relationships that combine competition and collaboration, assembly, disassembly, and reassembly, where key connections occur depending on the specific function of the volume, frequency, and the criticality of flows in a given period [2]. Alternatively, a ‘supply chain’ can be perceived as a complex socio-ecological system [3]. Major food supply chain disruptions can certainly cause food insecurity, as such acute disturbances affect all the pillars of food security, i.e., food availability, accessibility, utilisation, and stability [4,5]. The FAO states that:

*“A person is food insecure when they lack regular access to enough safe and nutritious food for normal growth and development and an active and healthy life. This may be due to unavailability of food and/or lack of resources to obtain food”*

[6].

The U.S. Department of Agriculture (USDA) points out that hunger is an individual-level physiological condition that may result from prolonged food insecurity [7]. A person who suffers from hunger feels uncomfortable and/or is in pain due to their insufficient consumption of dietary energy [6]. Supply chain resilience can reduce the likelihood of food shortages and assure some aspects of food security. Supply chain resilience refers to an organisation’s ability to recover from supply chain disruptions, where disruptions can be described as events that negatively influence planned product flow [8]. Supply chain disruptions can cause food loss, which may endanger food security, put pressure on the environment, and impede the sustainability of agri-food systems. Resilient supply chains may not operate at the highest level of resource efficiency or the lowest cost, but they are more capable of coping with the level of uncertainty in the business environments that they operate within [9]. Diversity in food supply chains aids coping strategies in the events of shocks and stresses as it provides multiple options to ensure resilience [10,11].

Under normal operational circumstances, implementing approaches, such as lean manufacturing, just-in-time inventory, the use of standardised components, and reductions in the supply base, reduces costs, facilitates greater efficiency, and improves financial margins [1,12]. Supply chain leanness has been shown to maximize profits through cost and waste reduction, while the ability to be agile, when needed, maximizes profit through providing exactly what the customer requires, especially where their requirements may change frequently [9]. Thus, it can be stated that both lean and agile management should result in food waste reduction, which means that less food is left unconsumed and/or discarded by retailers, which implies less risk to the environment and decreased risk of food insecurity. The following critical attributes of food supply chain management have been proposed: (1) relationship and governance; (2) coordination and integration; (3) collaboration; (4) agility; (5) logistics; (6) traceability; (7) packaging; and (8) waste management, and leanness and agility, to varying degrees, need to be embedded across all eight elements, along with other appropriate enabling systems and processes [13]. 

The food supply in developed markets is based on a ‘just-in-time’ operation, providing consumers with a wide range of safe, internationally sourced foods on a scale that drives efficiencies that can, in turn, reduce retail food prices. In theory, efficient markets should have been agile enough to minimize COVID-19 supply impacts; however, efficiency has driven reductionism, less redundancy, and reduced diversity, with the associated vulnerabilities. Indeed, the implementation of reductionist enterprise risk management (ERM) approaches with consideration of individual supply chain risks in isolation rather than aggregated risk, occurring in combination, or considering how they are influenced by multiple factors operating at different temporal loci means that supply chain risk, including food security risks, is often determined based on single failure points rather than as being affected by complex, interconnected, interdependent environmental, societal, and economic situations [14,15,16]. Thus, reductionist cost-driven lean approaches may reduce flexibility when unpredictable supply and demand patterns arise, so whilst consolidation and reductionism has led to supply chain efficiency, it has also created ‘pinch-points’ too.

Pinch points have been described as physical strategic points or locations in the supply chain which are bottlenecks, vulnerable to stress or shock, sensitive to disruption due to regulatory pressure, or have limited capacity to be agile now and in the future [17]. Bottlenecks and their impact on food security have been considered in terms of shocks, such as with Ukraine grain supplies [18], where agility was limited due to a lack of grain transport and a lack of capacity at grain export terminals. Emergency remedies to address this challenge included increasing logistics capacity and speeding up customs and phytosanitary procedures where a digital border control system was essential, with a better co-ordination of supply chain data (collection, aggregation, and dissemination). In a more proactive rather than reactive manner, there has been much research into the prediction of supply chain bottlenecks (see the study published by the authors of [19]), especially bottlenecks in fresh food supply in Nigeria [20], fresh pineapple supply in Benin [21], and bananas in Uganda [22]. An example of pinch points and potential vulnerability in developed food markets, for example, is that 95% of the United Kingdom (UK) food retail market is dominated by eight supermarkets [23]. Whilst risk can be mitigated, vulnerabilities and bottlenecks can still arise.

Limited capacity, at pinch points, can be formed as a result of a food safety or wider public health incident, political or social unrest, financial shock, or as a result of localised environmental failure, pest infestations, or business failure [17,24,25,26]. Operators in food supply chains can be vulnerable as they have always faced disruptions arising from many types of events, including natural disasters (e.g., floods, earthquakes, and hurricanes), pestilence (a fatal epidemic or pandemic disease affecting humans, livestock, or crops), insect and rodent plagues, man-made events (e.g., strikes and terrorist attacks), and industrial accidents (e.g., plant fires and food safety incidents), leading to potential food safety, supply, and quality problems e.g., adulterated food products [12,27]. Disruptions sparked by the COVID-19 pandemic resulted in the closure of numerous factories and led to the loss of key suppliers, and this problem increased as the impact of the pandemic elongated, disrupting supply chain relationships further [3]. In the early months of the COVID-19 pandemic, harvest, processing, and distribution pinch points were identified that led to the dumping of milk on farms, vegetables rotting in fields due to a lack of labour, along with food wastage due to consumer panic buying and hoarding [4,28,29]. Again, in the study published by Kumar et al. [28], digitalisation within supply chains was shown to be a mitigating, remedial strategy. Alabi and Ngwenyama [4] proposed such a framework that integrates system decentralisation, end-to-end supply chain visibility, application of Industry 4.0, cloud-based technology, and e-commerce platforms to deliver a smarter, more resilient global food system and food supply chains.

## 2. COVID-19: A Case That Demonstrates the Challenges Resulting from a Food Supply Chain Shock

The World Health Organisation (WHO) determined COVID-19 as a pandemic on 11 March 2020 [30], and global priority was given to mitigating the associated threat and the negative impact of the activities implemented to prevent the transmission of COVID-19 across a largely unvaccinated human population. Across the world, countries simply ‘locked-down’ in terms of restricting the movement of populations and in reducing activity within economies, with the resulting economic and social pressure. As a consequence of ‘locking down’, economies, and food supply chains in particular, faced major logistical disruptions. National ‘locking down policies’ were largely similar from country to country and included: education settings and workplace closure, or restricting workplace practices where locations were deemed essential, cancelling of public events, restrictions on personal and public gatherings more generally, and in homes, including shielding of vulnerable members of the population, stay-at-home requirements, restrictions on movement in countries, and international travel controls [31,32]. Indeed, Ketchen and Craighead [12] (p. 1335) asserted that “COVID-19 fuelled the most far-reaching and devastating supply chain disruptions in modern history”.

From a food supply perspective, the scale of the disruption was amplified by the fact that food systems are intrinsically complex, i.e., “*they comprise many different processes, value chains, actors and interactions; their outcomes affect multiple stakeholders and sectors in diverse and sometimes conflicting ways*” [33] (p. 17). In the ultra-lean modern food system, where supermarkets and other food retailers follow a ‘just-in-time’ approach, food supply chain stakeholders had to quickly reorganise/reconfigure to ensure the continued availability of food on retail shelves and the continued functioning of food supply chains [34,35]. Low stock holding of fruits and vegetables, for example by UK supermarkets, exacerbated by panic buying and disruption of European supply routes, created a vulnerability and as a result caused supply chain disruption [1].

In response to the COVID-19 pandemic, many countries imposed temporary restrictions on exports of certain foodstuffs (e.g., wheat, rice, maize, pulses, vegetable oil, eggs, lemons, onions, garlic, beans, milk powder, cheese, yogurt, beer, and spirits) in order to mitigate potential shortages of key supplies, peaking in March and April 2020. These countries were: Algeria, Argentina, Armenia, Belarus, Cambodia, Egypt, El Salvador, Gambia, Honduras, Kazakhstan, Kyrgyzstan, Moldova, Myanmar, North Macedonia, Pakistan, Romania, Russia, Serbia, Sudan, South Africa, Sudan, Syria, Tajikistan, Thailand, Turkey, Ukraine, and Vietnam [36]. These food export restrictions meant that about 5% of globally traded calories were affected [37]. The World Trade Organisation (WTO) called on members to consider the effects of restrictions on food security and to improve transparency on such trade measures [38]. Export restrictions contributed to reduced global food supply and caused rapid world food price increases by 12.9 percent on average in the quarter following the outbreak of the pandemic [39]. Imported food-dependent countries, including Tajikistan, Azerbaijan, Egypt, Yemen, and Cuba, were the most affected [39]. Such restrictions caused price volatility and induced uncertainty in food supply and reluctance to invest [38,40], and consequently posed threats to food availability and stability [41]. Furthermore, the analysis of the Global Food Security Index (GFSI) for the years 2020 and 2021 revealed that food export restrictions did not generally improve the food and nutrition security situation in the states that applied them [42].

**Figure 1:**
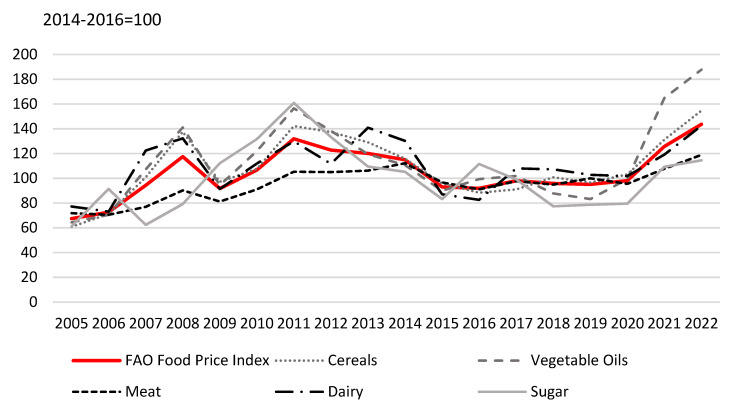
The FAO Food Price Index and FAO Food Commodity Price Indices from December 2005 to December 2022 (own elaboration based on the study published by the authors of [44]).

As in the 2007–2008 global economic crisis, decisions to introduce food export restrictions were justified by short-term food security concerns. Food export restrictions introduced during the financial crisis that started in 2007 contributed to constrain the upward trend of the food prices in the countries that applied them, but they also resulted in higher food prices in other countries as well [40,43]. Hence, it can be argued that food export restrictions introduced during the pandemic, and subsequently in the Ukraine/Russian conflict, have pushed international food prices up and further undermined the food security of the extremely poor, particularly in the aforementioned net-food-importing developing countries. The FAO Food Price Index rose in February and March 2022 due to the outbreak of war in Ukraine, but then fell markedly in the latter part of 2022, regarding, in particular, vegetable oils prices [44]. Thus, the overall index averaged 143.7 points in 2022, up by 14.3% from 2021, by 46.5% from 2020, and by 51.1% from 2019 [44] (Figure 1), which has had a negative impact on food accessibility in most countries. In order to protect developing countries, it has been argued that the agricultural export restriction policies should be more strictly regulated by the WTO negotiations even if the restrictions only exacerbate price spikes, since the UN sustainable development goal SDG1 defines a commitment “to leave no one behind and to reach those farthest behind first” [45].

### What Has Been the Impact on Food Security?

The FAO [46] (p. 1) stated that “while the global agri-food systems have remained resilient, income losses and food price spikes caused undernourishment to rise.” Indeed, the COVID-19 pandemic has contributed to the largest single-year increase in global hunger in decades. In 2020, there were 161 million more food-insecure people across the world than in 2019 [46]. The Ukraine/Russian conflict, which began in 2022, as previously described, has also disrupted the supply of grain, vegetable oil, fertiliser, and energy, leading to further food price rises [47]. The ongoing climate change and hardly predictable climate-related extreme weather events contribute to volatility in food production and prices and can push food prices up even further. Whilst global food prices peaked in March 2022, the global FAO Food Price Index is still 122 percent higher than 2014–16 levels compared with 160% in March 2022. In 2022, there were around 3.8 million fewer people suffering from hunger [44]. The FAO, IFAD, UNICEF, WFP, and WHO (2023) report states that around 29.6 percent of the global population (accounting for approximately 2.4 billion people) were moderately or severely food insecure in 2022, of which about 11.3 percent of the global population (equivalating to around 900 million people) were severely food insecure [48].

Agility through community action during lockdowns mitigated food insecurity for many families either through external intervention or internal household interventions, such as reducing portion size, but concerns over public health inequalities linked to poverty, especially in countries such as the UK, still remain [49,50,51]. Using a pro-market epistemology, it could be asserted that COVID-19 is a ‘once in a century event’ [52], or that conflict in Europe is an infrequent event, and as such the recent resilience of countries in terms of food security has ‘benefitted from a large degree of contingent luck’ [1] in terms of availability, but not with regard to affordability, a key element of food security. Increases in poverty and food insecurity during and after the COVID-19 pandemic have been cited in multiple countries, including Burkina Faso, Ethiopia, and Malawi [53]; Nigeria [53,54,55]; Canada and the United States (US) [4]; the UK [56]; Mexico and Bangladesh [54], among others. The literature reviewed in the next section reflects on the structure and operationalisation of food supply chains within food systems and how innate features of food supply chains create vulnerabilities and food insecurity. The concepts considered include leanness, agility, and the aggregated term, leagility.

## 3. Food Supply Chains: Structures and Operationalisation

The development of an organisational or supply chain strategy needs to specifically address the demand and supply factors that influence food systems and any specific constraints in ‘normal’ supply situations, and in the event of short-term or long-term geo-political and socio-economic shocks. The term resilience has been used throughout the narrative in this paper. Applying the resilience concept to food systems builds upon the considerations of food supply chains’ abilities to cope with external change in a way that does not undermine the food supply chain(s) and wider food system [57]. Thus, a resilient organisation in a food system produces safe food, ensuring the safety of its workers, minimises negative environmental and social externalities, and continuously seeks to develop its range of competences to cope with, economically survive, and succeed in a changing business environment. For a wider contemporary reflection on resilience see the study published by the authors of [58].

### 3.1. Leanness

The concept of lean manufacturing was first explained by the authors of [59] reporting findings of their study of the Toyota production system in Japan versus the American and European production strategies. Their lean methodology was mainly focused on the elimination of seven kinds of waste or ‘Muda’ in Japanese manufacturing, i.e., overproduction, waiting, transportation or conveyance, over processing or incorrect processing, excess inventory, unnecessary movements, and defects [60,61]. Lean thinking supports an approach to value that optimises resource use [62], where every activity that does not add value from the purchaser’s point of view is considered waste [63]. Thus, leanness can be described as doing more with less [64].

Implementing a lean strategy is particularly beneficial to organisations operating in a market where demand is relatively predictable with a low market variance (high certainty) and high volume [65]. Leanness is an efficiency strategy, exploiting human capital, the use of time and space, and the use of technology, infrastructure, and equipment. However, in more unpredictable markets where there is greater volatility, and higher levels of uncertainty, leanness may create a vulnerability, and even brittleness, where there is a lack of redundancy, agility, adaptive or buffer capacity to survive sudden changes, shocks, or squeezes [15]. The three major aspects of agility are involved suppliers, involved customers, and involved employees. These three aspects are also essential for an effective food safety management system within an organisation and across a food supply chain, especially ensuring employee involvement [66,67]. The adoption of lean thinking in business systems, and in particular food safety management, must go hand in hand [68], so that process improvement (being lean, more resilient, and less vulnerable) includes not only resource allocation and quality improvements, but also food safety management improvements. It is important to note that food safety and quality is one criteria within the GFSI along with affordability and availability, which have already been considered in this paper, and sustainability [42]. Addressing internal issues within the organisation, including continuous material flow, just-in-time systems with limited stock holding, setup/downtime time reduction, total productive/preventive maintenance, statistical process control, and employee involvement, are all essential to driving leanness through resource efficiency [68].

### 3.2. Agility

Agility, in this context, is a responsive strategy acting with speed to deliver business resilience [34,69]. Organisations are said to be agile if they can accommodate a variety of different kinds of change [70]. Being agile enables organisations to be resilient, and to grow in a volatile, competitive market, where they can adopt a rapid response to uncertain markets driven by customer-based evaluations of services and products [71,72,73]. Agility extends to the ability of people and/or autonomous systems to implement rapid and appropriate change within work processes, and for there to be sufficient visibility and connectivity to access the data required to inform evidence-based decision making when under pressure. Agility requires flexibility, interoperability (human–machine, and machine–machine), and the ability to be agile in problem detection and quantification, as well as in terms of workflow adaptability and responsiveness, or more simply, has aspects of detection, adaption, and responsiveness [74].

Building agility in organisational activities enhances flexible operations and processes, visibility through the supply chain, increased responsiveness, and higher market sensitiveness to dynamic demand. The concept of supply chain agility has evolved more recently from the nineties more in part due to the market turbulence caused by the COVID-19 pandemic. Consumers’ ability to be agile is also important in delivering food supply chain resilience; their ability to use different ingredients, break traditional habits, and embrace alternative food choices, as well as an ability to afford and have access to alternative ingredients and alternative supplies of food, e.g., food banks, will inform how consumption and purchasing patterns disrupt the resilience of food supply chains and food systems. The concept of supply chain agility states that the supply chain and/or individual businesses must possess both physical and cognitive capabilities to be alert, react quickly, and detect opportunities and threats; enact speedy and flexible actions; respond to all types of change initiated by both demand-side and supply-side market actors; and to deliver two main objectives: firstly to create competitive advantage and secondly, to effectively manage risks on an ongoing basis [34].

Substitutability is also an essential element of agility, for example, where human capital is employed, and when financial capital is under threat, to deliver a new organisational strategy or if certain physical resources are unavailable, employing human capital and associated physical capital (such as equipment and facilities) can support the business to adapt and identify new products and services that they can deliver. In crisis situations, developing agile rather than fragile food supply chains may require the substitution of unavailable products and services with alternatives with similar characteristics and performance (see the study published by the authors of [75] for an explanation of agility in the pharmaceutical supply chain). There are three distinct stages of the substitutability approach: pre-crisis, the crisis response, and the post-crisis phase [75]. In the pre-crisis phase (prevention and preparation), which is arguably the most important to focus on to ensure agility, the optimum stocks, stock maintenance conditions, and inventories are maintained. This strategy will interact with the lean focus of the organisation and the need for redundancy with some materials where higher stock holding may be required, e.g., engineering parts with a long lead time or materials that are essential to production but could be subject to supply chain disruption and/or where there is no recognised option for substitutability. Some of these items may be needed at critical control points and are essential for effectively implementing the food safety management plan. The crisis phase captures the detection processes, the management response to network disruption, and the work process adaptability to deliver an efficient, agile, flexible, and resilient response. The third phase post-crisis is the management reflection and feedback mechanisms that may capture the elements of problem mapping, problem analysis, and redesign elements, as proposed by Jacxsens et al. [76]. 

### 3.3. Leagility

Leagility is positioned as the evolution from lean thinking to the agile paradigm [77,78]. Promoting the concept of leagility in supply chain management links the concepts of lean and agility to resilience (survival and recovery) and sustainable practices, i.e., supply chain strategic requirements, such as delivering net zero greenhouse gas emissions, addressing climate change, and embedding the sustainable development paradigm, often through digitalisation [79]. Where product demand is constant and stable and can be met with existing supply and procurement activities, albeit with some substitutability if needed, then lean practices can be adopted. However, if demand or supply dynamics become more unpredictable then the organisational need to be more agile increases. Supply chain strategies towards leanness and agility can be developed on the basis of the types of products produced and the particular supply and demand dynamics of the supply chain and the potential for supply chain shocks to occur. Thus, while functional products require an efficient and lean supply chain with a cost reduction approach, conversely, innovative products need a more agile, responsive, and flexible supply chain with a high delivery speed [80].

Lean thinking and agile manufacturing paradigms cannot be developed in isolation and organisations should carefully combine both lean and agile paradigms in their organisational strategy [81]. Naylor et al. [81] referred to the notion of the ‘decoupling point’, which separates the aspects of the supply chain orientated towards customer orders from the elements of supply chain planning, stating that the decoupling point is “the point at which strategic stock is often held as a buyer between fluctuating customer orders and/or product variety and smooth production output” [81] (p. 108). In essence, the decoupling point separates the lean aspects of strategy from the agile, and there may be times where capacity management or resource allocation is focused on leanness, and other times where the organisational focus is on agility, or leanness and agility at the same time [64]. Decoupling points can relate to materials [82], customer orders [83], and a push-pull boundary between raw material procurement and production or between production and distribution [84]. As Amir [85] (p. 287) stated:

*“Downstream from the decoupling point all products are pulled by the customer demand, that is why that part of supply chain is market driven. Upstream from the decoupling point the supply chain is essentially forecast driven”.*

This hybrid concept is termed ‘leagility’. Thus, the leagile supply chain is a hybrid paradigm in which leanness and agility are implemented in upstream and downstream supply chains, respectively [64,86], and fifteen proposed critical leagile enablers have been drawn together in Table 1. The trade-offs between lean, agile, resilient, and green (LARG) food supply chain management paradigms are important in both non-crisis ‘business-as-usual’ and in crisis situations, especially where they impact on food security. In this context, a paradigm is defined as a way of explaining a phenomenon or situation, or a dominant viewpoint of a given construct e.g., a food supply chain or the totality of the food system. LARG management paradigms can help supply chains to become more efficient, streamlined, and sustainable [9]. Indeed, there can be integration of lean, agile, resilient, green, and sustainable (LARGS) paradigms within the supply chain domain [87].

The question that arises when analysing the effects of the COVID-19 pandemic on food supply chain resilience, and in particular supply chain agility, is whether the problems that are being witnessed at local, national, and global scales result solely from the shock of the COVID-19 pandemic, and the associated mitigation strategies that have been implemented to reduce viral transmission, or whether these problems arise from more long-term systemic problems in how food supply chains are structured and then operationalised within the wider food system. For example, during a supply system shock, individuals can perceive future resources as scarcer than they are in reality (see the study published by the authors of [91] concentrating on food resources in this context). Resource scarcity can cause a sharp supply decrease, putting pressure on the demand side of the business [92] and potentially shifting the decoupling point. The impact on actual food supplies can also seem perverse. For instance, there is generally only around 10 days’ availability of food mobilised in the UK supply chain, less for perishables, and typically only 1.5 days for Spanish citrus fruits with logistical pinch points being key seaports, such as Dover [93]. Indeed, there is evidence to suggest that not only did prices of imported fruits and vegetables rise in the UK, but that actual import volumes of tomatoes, onions, and grapes reduced in the first quarter of 2020 [94]. However, it is difficult to quantify whether this was solely due to the impact of the COVID-19 pandemic, or the dual supply chain shocks of Brexit and COVID-19 combined.

### 3.4. Viability

Implementing leanness and maximising return on resources requires the mapping of the supply chain(s), especially pinch points and decoupling points, which may or may not be synonymous, and undertaking effective risk reduction strategies. Supply chain mapping will also identify where there are potential areas of resource waste to enable interventions to eliminate or mitigate them by putting appropriate strategies in place. Whilst the aim of leanness is to minimise redundancy via a just-in-time approach, and to ensure low levels of financial capital are embedded in physical stock and a reduction in labour costs, this can reduce resilience in the event of an unexpected shock or event [15]. However, a single approach to supply chain management creates vulnerabilities. Whilst leanness, e.g., minimising over supply or product/service failure, may reduce waste and optimise resource efficiency, alternatively, agility delivers process optimisation in a fast changing market and wider environment [85]. A viable supply chain has been defined by Ivanov [79] (p. 1415) as:

*“a dynamically adaptable and structurally changeable value-adding network able to (i) react agilely to positive changes, (ii) be resilient to absorb negative events and recover after the disruptions, and (iii) survive at the times of long-term, global disruptions by adjusting capacities utilizations and their allocations to demands in response to internal and external changes in line with the sustainable developments to secure the provision of society and markets with goods and services in long-term perspective”.*

Supply chain viability implies a long-term maintenance of survivability under changing conditions [95]. Here, viability can be considered as an underlying supply chain property spanning three perspectives, i.e., agility, resilience, and sustainability [79]. The viable supply chain model proposed by Ivanov [79] provides guidance to decision makers in a company regarding recovery and rebuilding of supply chains during and after global, massive, and long-term crises, such as the COVID-19 pandemic.

## 4. Discussion

**Figure 2:**
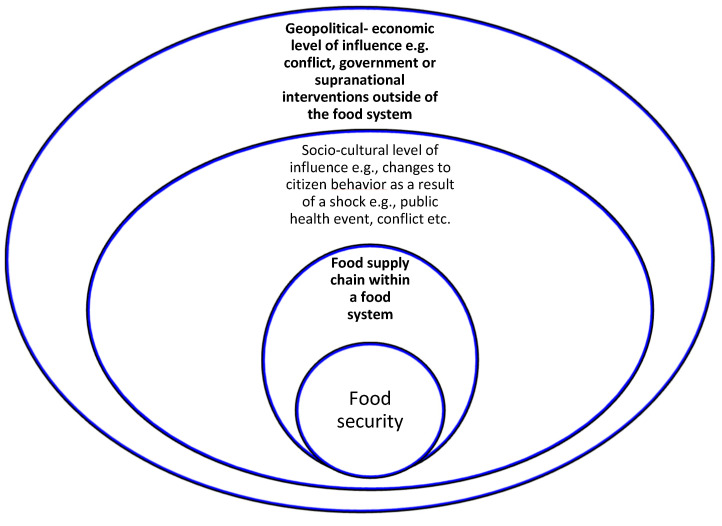
The socio-technical levels of food supply chains.

The COVID-19 pandemic provides insight into the entanglement of a food supply chain and the food system with other systems that operate at different socio-technical levels, i.e., firstly, at the **geo-political-economic level**, where politicians imposed the closure of factories, restaurants, and schools to curb the spread of SARS-CoV-2; secondly, at the **socio-cultural level**, where the pandemic resulted in novel food consumption patterns; and finally, at the **supply chain level**, where food supply chain managers began to question the benefits, but also the problems associated with overreliance on single sourcing of materials and ingredients, leading to a lack of resilience in the enacting of inventory-based procurement policies. Strategic management must focus on the complexity of a safe, secure, and agile food supply extending beyond the consideration of the structure, inventory, and interaction of elements of the food supply chains to also assess the influence of geo-political-economic and socio-cultural drivers in a given situation (Figure 2). 

Some argue that the COVID-19 pandemic demonstrated that food systems are broken [96], but this assertion was primarily positioned through the lens of health inequalities and their relationship to the food system. Some studies suggest that government policy responses to the COVID-19 pandemic exposed households to reducing incomes in both developed and developing countries [41,97,98], resulting in a limited ability to buy adequate, nutritious food. However, lockdowns or restrictions on movement have varied substantially by country and, consequently, the scale of the problem of income loss has been different across the world. Unsurprisingly, at the start of the COVID-19 pandemic, the WTO, USAID, and the OECD focused on opening international markets and encouraging global trade, stating that the global system was very prepared and resilient, and in a strong position to respond to the COVID-19 crisis, citing ‘open and predictable’ trading has ensured that food is moved to where it is required, meaning that it is accessible [35]. However, having food on supermarket shelves does not necessarily provide a sufficient response to hunger for some sub-groups of a population [23,99]. Indeed, the impact of COVID-19 on global food systems cannot be analysed in isolation, when so many underlying vulnerability factors can still be attributed to the legacy of the 2007–2008 global financial crisis. Research commissioned by the UK Food Standards Agency on the impact of the pandemic on food consumers’ behaviours and preferences in 2020 [100] highlighted a rise in food insecurity caused by physical and financial barriers to purchasing food as a result of the pandemic and the implementation of mitigation measures (various forms of lockdown and self-isolation, which brought severe losses of income). Following the 2007–2008 financial crisis, there was a rise in food aid in both developed and developing countries. Indeed, the rise in numbers of food banks in developed economies and more requests for help to access food suggests a normalising of food aid [101,102]. In what has been described in the UK as a ‘public health emergency’ long before COVID-19 [103], the lack of continuous access to a nutritious diet has been attributed to ongoing financial insecurity.

Some argue that the instigation and overutilisation of food banks create a dependency culture on foods that are often of a poor nutritional quality when part of a long-term diet [104], and thus create a vulnerability in the event of a subsequent public health and/or supply chain shock. The demand for food aid rose significantly at the start of the COVID-19-related lockdown in March 2020 and retail food supply chains struggled to fill the food gap. The number of people facing food insecurity was estimated to have quadrupled to ca. 8 million in the UK in April 2020 [50]. Despite Defra providing around £16 million to aid in food redistribution [105], this still equates to 16% of the population being food insecure [49]. This arguably associates COVID-19 with a rise in food insecurity and demonstrates the impact the pandemic has had on the geopolitical-economic environment framing food insecurity in the UK and more widely. The COVID-19 pandemic has drawn attention to the geopolitical-economic and socio-cultural issues framing food insecurity, forcing governments to seek short-term, reactive, and piecemeal remedies [56], focusing especially on food supply to vulnerable members of the community [105].

**Figure 3:**
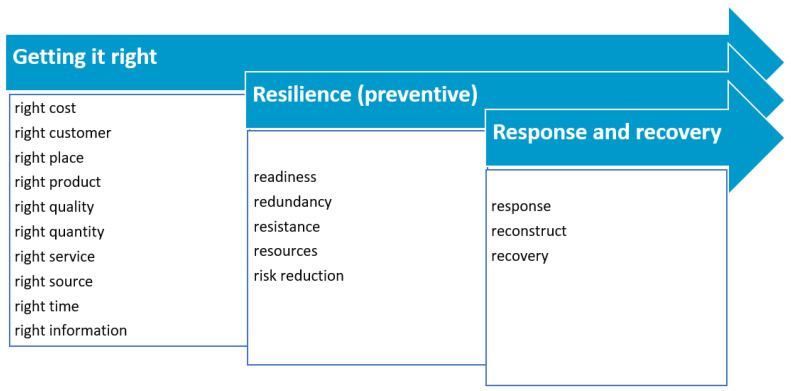
The R’s of supply chain resilience (adapted from the works published by the authors of [107,108]).

Given that supply chains have experienced difficult geopolitical-economic disruptions during the COVID-19 pandemic, and now more recently with the Ukraine/Russian conflict, the work of Ivanov [79] considered the interaction between leagility, resilience, sustainability, digitalisation, and viability, and the proposition of a viable supply chain model to serve the development of novel approaches to organisational and supply chain level decision making is of contemporary interest. The aim of leanness is to deliver the 8Rs of logistics in the digital age (Industry 4.0) [106] extended by others [85] to 10Rs which are as follows: right cost, right customer, right product, right place, right quality, right quantity, right service, right source, right time, and right information. In terms of agility, adaptive capacity, and resilience, five further Rs can be added, which are: resources, risk reduction, readiness, response and recovery [107], and redundancy and resistance. In this context of a global crisis, resilience from a macroeconomic perspective comprises an ability to limit immediate production losses and to ‘reconstruct and recover’ [108]. The R’s of supply chain resilience have been captured in Figure 3.

The COVID-19 pandemic has shown that where previously, predictability and certainty were assumed characteristics across the food supply chain when developing lean approaches, these characteristics are not assured in a crisis, meaning effective contingency planning must also be adopted. Resilience is a central perspective, reducing brittleness and promoting supply chain viability in the short, middle, and long term. While resilience can be perceived as being able to withstand and recover from disruption [79], supply chain resilience, and as a result food security, is enacted when the supply network is “capable to withstand, adapt, and recover from disruptions to meet customer demand and ensure performance” [109] (p. 285). Such an approach to resilience has been also supported by O’Meara et al. [110] (p. 100594) who stated that:

*“resilience it is not merely about withstanding stressors and shocks but more importantly the ability to build capacity to anticipate, prevent, absorb, and adapt from these experiences”.*

In this context, De Steenhuijsen Piters et al. [111] have identified four key resilience properties of food systems and the food supply chains that operate within them, and they called it “the ABCD of resilience building”, i.e.,:

**Agency** (the means and capacities of people to mitigate risks and to respond to change, disruptions, and crises); 

**Buffering** (resources to fall back on in the face of shocks and stressors, e.g., financial support to individuals and businesses and national food stocks, which have been defined as buffer capacity by others (see the studies published by the authors of [112,113,114]));

**Connectivity** (the interconnection of, and communication between, actors in the agri-food value chains and market segments); 

**Diversity** in the entire food system, including production, consumption, economy, governance, and society, which means that one resource may be quite easily replaced by another at different scales and in different places, from production to consumption and from farm level to regional diversity.

It is necessary to build resilience and food security into the food system at multiple scales: at micro (household), meso (community), and macro (national and international) levels to promote agility and minimise fragility, but this may come at a cost. The hybrid concept leagility is a paradigm that focuses on the elimination of waste and non-value-added activities across the food supply chain, and, at the same time, it enables the ability to meet market requirements, requiring buffer and adaptive capacity. Leagility is also a mechanism that can assist in pre-, during, and post crisis situations, and also deliver sustainable growth with a focus on the three pillars of secure and sustainable food systems: economic, environmental, and social sustainability. The difference between a resilience-focused approach compared to a more conventional food risk management approach is that:

*“with resilience the key is the ability to adapt [adaptive capacity] while the goal of conventional risk management approach is to resist (i.e., prevent or eliminate) [resistance capacity] food safety shocks”*

[115] (p. 5).

Therefore, further socio-economic disruptions or unpredictable events can mean existing risk assessment processes may fail to deliver appropriate resilient strategies, especially where the extent of the shock may be so great that with the lean measures adopted there is an inability to resist or buffer against the impact. This difference between adaptive capacity and resistance capacity is very important when considering food security at household through to global levels and is worthy of further research. Whilst adaptive capacity is considered within the literature in terms of food security, for example, in Kenya [116,117] and Ethiopia [118], among others, resistance capacity has not been widely studied. The work of Folke et al. [119] suggested that food security resilience needs to be considered in terms of adaptive capacity, resistance capacity, i.e., surviving shocks whilst retaining existing structures and functionality, and transformative capacity to transition to new models of operation.

Research focused on the impact of the COVID-19 pandemic in Europe [120] and in African countries [121] showed both common and different effects in developed and emerging economies. The impact on all nations has been profound, potentially setting back the global delivery of the Sustainable Development Goals (SDGs) by 2030, including zero hunger and poverty reduction, and those related to climate change [122]. In summary, the COVID-19 pandemic has exposed a number of economic and social vulnerabilities in both global and national food systems [123], and this crisis affords the opportunity to reflect on the overall resilience of contemporary food systems [1], and within them specific food supply chains and their ability to deliver a safe, secure food supply at all levels and in all locations. 

## 5. Conclusions

Being lean has advantages for an organisation and/or supply chain, but overemphasis on creating a just-in-time strategy in the absence of organisational or supply chain enablers that can deliver agility can lead to brittleness, fragility, and ultimately food insecurity. The inability to react to a food safety or food security risk when it arises due to a lack of redundancy with limited resources (financial, human, physical, etc.) is one example of such brittleness. Agility underpins resilience, which should extend beyond coping, surviving, and maintaining viability to ensure that food organisations and supply chains can grow and evolve, especially during periods of rapid change, volatility, and uncertainty. In recent times, the functioning of the global food supply chains was disrupted with geo-political and socio-economic shocks associated with the pandemic and the war in Ukraine, but also with extreme weather events and natural disasters driven by global warming. Developing smart food supply chains with the use of the newest technology (e.g., artificial intelligence, distributed ledger technologies, Internet of Things, and so on) creates vulnerability to cyber attacks and requires the continuous development of shock tactics. This review supports a critique of the strengths and vulnerabilities that have been identified in food systems in order to propose more resilient structures that could be adopted to reduce vulnerability to supply chain shocks, which will certainly arise in the future, and ensure the safety and security of food supply chains. Leagility is one particular approach.

## Figures and Tables

**Table 1:** Critical leagile enablers (adapted from the study published by the authors of [88]).

Leagile Enabler	Description
**Dependent enablers with strong dependence and weak driving power**	
Corporate culture	Corporate culture needs to be focused on customer orientation, continuous improvement, employee empowerment, and evidence-based decision making.
Flexibility	A leagile supply chain needs to be flexible and responsive to all market and geo-political conditions.
Knowledge management	Effective processes for acquiring, creating, and sharing knowledge within and between organisations, and also effectively managing organisational forgetting, unlearning, learning, and relearning [89,90].
Risk reduction	Improved risk identification, assessment, management, and response through collaboration, knowledge sharing, and core competencies is essential.
Strategic management	Creating and sustaining competitive advantage and strategic management is essential to leagility.
Virtualisation	Creating agility through being a virtual organisation that can respond quickly to business opportunities. Virtual supply chains are information centred rather than inventory centred [85].
**Independent enablers with weak dependence and strong driving power**	
Adaptability	Adaptability is a key enabler in uncertain and volatile markets and environments.
Cycle time reduction measures	Reducing cycle times enables responsiveness to customer needs and improves competitiveness, performance, and economic returns.
Just-in-time	The elimination of unnecessary activities, waste, or time delays. However, a just-in-time approach can also create risks that need to be effectively mitigated.
Positioning of decoupling point	Identifying the point where leanness and agility need to be integrated and differentiated.
Relatedness	Supply chain relationships need to enable responsiveness through collaboration and knowledge sharing.
Responsiveness	Rapid effective reactions to change and demands. Responsiveness integrates virtualisation, information integrity, competencies, and knowledge management.
**Autonomous enablers with weak dependence and weak driving power**	
Customer and market sensitivity	Digitalisation can improve closeness through access to customer data and improve accuracy, enable new process and product development, enable effective change management, and economic returns.
Information integrity	Accurate information and a minimisation of errors.
Training, skills, and competencies development in people	Developing the knowledge, skills, and competencies of individuals and teams to achieve leagility.

## Data Availability

Not applicable.

## References

[1] Garnett P., Doherty B., Heron T. (2020). Vulnerability of the United Kingdom’s food supply chains exposed by COVID-19. Nat. Food.

[2] Hearnshaw E.J.S., Wilson M.M.J. (2013). A complex network approach to supply chain network theory. Int. J. Oper. Prod. Manag..

[3] Wieland A. (2021). Dancing the Supply Chain: Toward Transformative Supply Chain Management. J. Supply Chain Manag..

[4] Alabi M.O., Ngwenyama O. (2023). Food security and disruptions of the global food supply chains during COVID-19: Building smarter food supply chains for post COVID-19 era. Br. Food J..

[5] Kowalski J., Kowalska A. (2022). The realization of the human right to food: Preliminary remarks on assessing food security. Przegląd Prawno-Ekon..

[6] FAO Hunger and Food Insecurity. https://www.fao.org/hunger/en/.

[7] USDA Economic Research Service Definitions of Food Security. https://www.ers.usda.gov/topics/food-nutrition-assistance/food-security-in-the-us/definitions-of-food-security/.

[8] Craighead C.W., Blackhurst J., Rungtusanatham M.J., Handfield R.B. (2007). The severity of supply chain disruptions: Design characteristics and mitigation capabilities. Decis. Sci..

[9] Carvalho H., Duarte S., Machado V.C. (2011). Lean, agile, resilient and green: Divergencies and synergies. Int. J. Lean Six Sigma.

[10] Berkes F. (2007). Understanding uncertainty and reducing vulnerability: Lessons from resilience thinking. Nat. Hazards.

[11] Darnhofer I. (2010). Strategies of family farms to strengthen their resilience. Environ. Policy Gov..

[12] Ketchen D.J., Craighead C.W. (2020). Research at the intersection of entrepreneurship, supply chain management, and strategic management: Opportunities highlighted by COVID-19. J. Manag..

[13] Sufiyan M., Haleem A., Khan S., Khan M.I., Shanker K., Shankar R., Sindhwani R. (2019). Analysing Attributes of Food Supply Chain Management: A Comparative Study. Advances in Industrial and Production Engineering. Lecture Notes in Mechanical Engineerin.

[14] Pettit T.J., Croxton K.L., Fiksel J. (2019). The evolution of resilience in supply chain management: A retrospective on ensuring supply chain resilience. J. Bus. Logist..

[15] Manning L., Birchmore I., Morris W. (2020). Swans and elephants: A typology to capture the challenges of food supply chain risk assessment. Trends Food Sci. Technol..

[16] Kowalska A., Manning L. (2022). Food Safety Governance and Guardianship: The Role of the Private Sector in Addressing the EU Ethylene Oxide Incident. Foods.

[17] Soon J.M., Manning L., Smith R. (2019). Advancing understanding of pinch-points and crime prevention in the food supply chain. Crime Prev. Community Saf..

[18] Semenenko O., Tolok P., Onofriichuk A., Onofriichuk V., Chernyshova I. (2023). Improving Ukrainian grain export supply chains: An inclusive approach. Int. J. Environ. Stud..

[19] Mizgier K.J., Jüttner M.P., Wagner S.M. (2013). Bottleneck identification in supply chain networks. Int. J. Prod. Res..

[20] Onwude D., Motmans T., Shoji K., Evangelista R., Gajardo J., Odion D., Ikegwuonu N., Adekanmbi O., Hourri S., Defraeye T. (2023). Bottlenecks in Nigeria's fresh food supply chain: What is the way forward?. Trends Food Sci. Technol..

[21] Fassinou Hotegni V.N., Lommen W.J., van der Vorst J.G., Agbossou E.K., Struik P.C. (2014). Bottlenecks and opportunities for quality improvement in fresh pineapple supply chains in Benin. Int. Food Agribus. Manag. Rev..

[22] Ssennoga F., Mugurusi G., Oluka P.N. (2019). Food insecurity as a supply chain problem. Evidence and lessons from the production and supply of bananas in Uganda. Sci. Afr..

[23] Cooper K., Birmingham Food Council (2020). COVID-19 Commentary: 13 Features of Our Pre-COVID Food System. https://www.birminghamfoodcouncil.org/2020/04/30/covid-19-commentary-13-features-of-our-pre-covid-food-system/.

[24] Christopher M., Peck H. (2004). Building the resilient supply chain. Int. J. Logist. Manag..

[25] Yakovleva N., Flynn A. (2004). Innovation and sustainability in the food system: A case of chicken production and consumption in the UK. J. Environ. Policy Plan..

[26] Makwasha T., Turner B. (2013). Evaluating the use of rural-urban gateway treatments in New Zealand. J. Australas. Coll. Road Saf..

[27] Manning L. (2021). Safeguard global supply chains during a pandemic. Nat. Food.

[28] Kumar V., Yetkin Ekren B., Wang J., Shah B., Frederico G.F. (2023). Investigating the impact of COVID-19 on sustainable food supply chains. J. Model. Manag..

[29] Rahimi P., Islam M.S., Duarte P.M., Tazerji S.S., Sobur M.A., El Zowalaty M.E., Ashour H.M., Rahman M.T. (2022). Impact of the COVID-19 pandemic on food production and animal health. Trends Food Sci. Technol..

[30] WHO (2020). WHO Director-General’s Opening Remarks at the Media Briefing on COVID-19—11 March 2020. https://www.who.int/director-general/speeches/detail/who-director-general-s-opening-remarks-at-the-media-briefing-on-COVID-19---11-march-2020.

[31] Hale T., Angrist N., Goldszmidt R., Kira B., Petherick A., Phillips T., Webster S., Cameron-Blake E., Hallas L., Majumdar S. (2021). A global panel database of pandemic policies (Oxford COVID-19 Government Response Tracker). Nat. Hum. Behav..

[32] Ritchie H., Mathieu E., Rodés-Guirao L., Appel C., Giattino C., Ortiz-Ospina E., Hasell J., Macdonald B., Diana Beltekian D., Roser M. (2021). Coronavirus Pandemic (COVID-19). https://ourworldindata.org/coronavirus.

[33] Tendall D.M., Joerin J., Kopainsky B., Edwards P., Shreck A., Le Q.B., Kruetli P., Grant M., Six J. (2015). Food system resilience: Defining the concept. Glob. Food Secur..

[34] Do Q.N., Mishra N., Wulandhari N.B.I., Ramudhin A., Sivarajah U., Milligan G. (2021). Supply chain agility responding to unprecedented changes: Empirical evidence from the UK food supply chain during COVID-19 crisis. Supply Chain. Manag..

[35] OECD (2020). Food Supply Chains and COVID-19: Impacts and Policy Lessons. http://www.oecd.org/coronavirus/policy-responses/food-supply-chains-and-covid-19-impacts-andpolicy-lessons-71b57aea/#abstract-d1e27.

[36] OECD (2021). COVID-19 and Food Systems: Short- and Long-Term Impacts. OECD Food, Agriculture and Fisheries Papers.

[37] Hepburn J., Laborde D., Parent M., Smaller C. (2020). COVID-19 and Food Export Restrictions: Comparing Today’s Situation to the 2007/08 Price Spikes. https://www.iisd.org/system/files/2020-08/covid-19-food-export-restrictions.pdf.

[38] Congressional Research Service (2021). Export Restrictions in Response to the COVID-19 Pandemic. https://crsreports.congress.gov/product/pdf/IF/IF11551.

[39] Espitia A., Rocha N., Ruta M. (2020). COVID-19 and Food Protectionism: The Impact of the Pandemic and Export Restrictions on World Food Markets. World Bank Policy Res. Work. Pap..

[40] Laborde D., Estrades C., Bouët A. (2013). A Global Assessment of the Economic Effects of Export Taxes. World Econ..

[41] Laborde D., Martin W., Swinnen J., Vos R. (2020). COVID-19 risks to global food security. Science.

[42] Kowalska A., Budzyńska A., Białowąs T. (2022). Food export restrictions during the COVID-19 pandemic: Real and potential effects on food security. Int. J. Manag. Econ..

[43] Anania G., Méndelez-Ortiz R., Bellman C., Hepburn J. (2014). Export restrictions and food security. Tackling Agriculture in the Post-Bali Context. A Collection of Short Essays.

[44] FAO, IFAD, UNICEF, WFP, WHO (2022). The State of Food Security and Nutrition in the World 2022. Repurposing Food and Agricultural Policies to Make Healthy Diets More Affordable.

[45] United Nations Development Programme (UNDP) (2016). Leaving No One Behind: A Social Protection Primer for Practitioners. https://www.undp.org/publications/leaving-no-one-behind-social-protection-primer-practitioners.

[46] FAO (2021). FAO’s Response to COVID-19: Building to Transform. https://www.fao.org/3/ng635en/ng635en.pdf.

[47] FAO (2023). World Food Situation. FAO Food Price Index. https://www.fao.org/worldfoodsituation/foodpricesindex/en/.

[48] FAO, IFAD, UNICEF, WFP, WHO (2023). The State of Food Security and Nutrition in the World 2023. Urbanization, Agrifood Systems Transformation and Healthy Diets across the Rural–Urban Continuum.

[49] Loopstra R. (2020). Vulnerability to Food Insecurity Since the COVID-19 Lockdown Preliminary Report. https://foodfoundation.org.uk/sites/default/files/2021-10/Report_COVID19FoodInsecurity-final.pdf.

[50] The Food Foundation (2020). New Food Foundation Survey: Three Million Britons Are Going Hungry Just Three Weeks into Lockdown. https://foodfoundation.org.uk/new-food-foundation-survey-three-million-britons-are-going-hungry-just-three-weeks-into-lockdown/.

[51] Macaninch E., Martyn K., Lima do Vale M. (2020). Exploring the implications of COVID-19 on widening health inequalities and the emergence of nutrition insecurity through the lens of organisations involved with the emergency food response. BMJ Nutr. Prev. Health.

[52] The Economist Keeping Things Cornucopious—The World’s Food System Has so Far Weathered the Challenge of COVID-19, Briefing, The Economist. 9 May 2020. https://www.economist.com/briefing/2020/05/09/the-worlds-food-system-has-so-far-weathered-the-challenge-of-covid-19.

[53] Rudin-Rush L., Michler J.D., Josephson A., Bloem J.R. (2022). Food insecurity during the first year of the COVID-19 pandemic in four African countries. Food Policy.

[54] Picchioni F., Goulao L.F., Roberfroid D. (2022). The impact of COVID-19 on diet quality, food security and nutrition in low and middle income countries: A systematic review of the evidence. Clin. Nutr..

[55] Balana B.B., Ogunniyi A., Oyeyemi M., Fasoranti A., Edeh H., Andam K. (2023). COVID-19, food insecurity and dietary diversity of households: Survey evidence from Nigeria. Food Secur..

[56] Barker M., Russell J. (2020). Feeding the food insecure in Britain: Learning from the 2020 COVID-19 crisis. Food Secur..

[57] Ericksen P., Bohle H.-G., Stewart B., Ingram J., Ericksen P., Liverman D. (2010). Vulnerability and Resilience of Food Systems. Food Security and Global Environmental Change.

[58] Manning L. (2023). Being resilient in challenging times in food supply chains. Presented at The International Conference on Industry 4.0 for Agri-food Supply Chains: Addressing: Socio-economic and Environmental Challenges in Ukraine, Leicester, United Kingdom, 24–25 July 2023. Eng. Proc..

[59] Womack J., Jones D., Roos D. (1990). The Machine that Changed the World: The Story of Lean Production, Toyota’s Secret Weapon in the Global Car Wars That Is Now Revolutionizing World Industry.

[60] Ohno T. (1988). Toyota Production Systems: Beyond Large Scale Production.

[61] Bhamra R., Nand A., Yang L., Albregard P., Azevedo G., Corraini D., Emiliasiq M. (2021). Is leagile still relevant? A review and research opportunities. Total Qual. Manag. Bus. Excell..

[62] Vakadae Ramkumar U., Raja M.S., Varghese J. (2021). Organisational Agility and 7Ps of the marketing mix for the post-COVID-19 period: A case study of the Indian informal food sector. J. Contemp. Issues Bus. Gov..

[63] Bicheno J.R., Holweg M. (2004). The New Lean Toolbox.

[64] Rahimnia F., Moghadasian M. (2010). Supply chain leagility in professional services: How to apply decoupling point concept in healthcare delivery system. Supply Chain Manag. Int. J..

[65] Christopher M.C. (2000). The agile supply chain: Competing in volatile markets. Ind. Mark. Manag..

[66] Taha S., Wilkins S., Juusola K., Osaili T.M. (2020). Food safety performance in food manufacturing facilities: The influence of management practices on food handler commitment. J. Food Prot..

[67] Wilcock A., Ball B., Fajumo A. (2011). Effective implementation of food safety initiatives: Managers’, food safety coordinators’ and production workers’ perspectives. Food Control.

[68] Dora M., Kumar M., Van Goubergen D., Molnar A., Gellynck X. (2013). Operational performance and critical success factors of lean manufacturing in European food processing SMEs. Trends Food Sci. Technol..

[69] Nandi S., Sarkis J., Hervani A., Helms M. (2020). Do blockchain and circular economy practices improve post COVID-19 supply chains? A resource-based and resource dependence perspective. Ind. Manag. Data Syst..

[70] Dove R. (1996). Agile supply-chain management. Automot. Prod..

[71] Jain V., Kumar S., Soni U., Chandra C. (2017). Supply Chain Resilience: Model Development and Empirical Analysis. Int. J. Prod. Res..

[72] Tukamuhabwa B., Stevenson M., Busby J. (2017). Supply Chain Resilience in a Developing Country Context: A Case Study on the Interconnectedness of Threats, Strategies and Outcomes. Supply Chain. Manag. Int. J..

[73] Yusuf Y., Sarhadi M., Gunasekaran A. (1999). Agile manufacturing: The drivers, concepts and attributes. Int. J. Prod. Econ..

[74] Barthe-Delanoë A.M., Truptil S., Bénaben F., Pingaud H. (2014). Event-driven agility of interoperability during the Run-time of collaborative processes. Decis. Support Syst..

[75] Roshan M., Tavakkoli-Moghaddam R., Rahimi Y. (2019). A two-stage approach to agile pharmaceutical supply chain management with product substitutability in crises. Comput. Chem. Eng..

[76] Jacxsens L., Luning P.A., Marcelis W.J., van Boekel T., Rovira J., Oses S., Kousta M., Drosinos E., Jasson V., Uyttendaele M. (2011). Tools for the performance assessment and improvement of food safety management systems. Trends Food Sci. Technol..

[77] Kisperska-Moron D., De Haan J. (2011). Improving supply chain performance to satisfy final customers: “leagile” experiences of a Polish distributor. Int. J. Prod. Econ..

[78] Qi Y., Huo B., Wang Z., Yeung H.Y.J. (2017). The impact of operations and supply chain strategies on integration and performance. Int. J. Prod. Econ..

[79] Ivanov D. (2022). Viable supply chain model: Integrating agility, resilience and sustainability perspectives—Lessons from and thinking beyond the COVID-19 pandemic. Ann. Oper. Res..

[80] Fisher M. (1997). What is the right supply chain for your product?. Harv. Bus. Rev..

[81] Naylor J.B., Naim M.M., Berry D. (1999). Leagility: Integrating the lean and agile manufacturing paradigms in the total supply chain. Int. J. Prod. Econ..

[82] Mason-Jones R., Towill D.R. (1999). Using the information decoupling point to improve supply chain performance. Int. J. Logist. Manag..

[83] Olhager J. (2010). The role of the customer order decoupling point in production and supply chain management. Comput. Ind..

[84] Jeong I.J. (2011). A dynamic model for the optimization of decoupling point and production planning in a supply chain. Int. J. Prod. Econ..

[85] Amir F. (2011). Significance of lean, agile and leagile decoupling point in supply chain management. J. Econ. Behav. Stud..

[86] Mason-Jones R., Naylor B., Towill D.R. (2000). Lean, agile or leagile? Matching your supply chain to the marketplace. Int. J. Prod. Res..

[87] Sharma V., Raut R.D., Mangla S.K., Narkhede B.E., Luthra S., Gokhale R. (2021). A systematic literature review to integrate lean, agile, resilient, green and sustainable paradigms in the supply chain management. Bus. Strategy Environ..

[88] Tamtam F., Tourabi A. (2021). Interpretive structural modeling of supply chain leagility during COVID-19. IFAC-PapersOnLine.

[89] Manning L., Morris W., Birchmore I. (2021). Organisational forgetting: The food safety risk associated with unintentional knowledge loss. Trends Food Sci. Technol..

[90] Manning L., Morris W., Birchmore I. (2023). Organizational unlearning: A risky food safety strategy?. Compr. Rev. Food Sci. Food Saf..

[91] Folwarczny M., Li N.P., Sigurdsson V., Tan L.K.L., Otterbring T. (2021). Development and psychometric evaluation of the Anticipated Food Scarcity Scale (AFSS). Appetite.

[92] Shaheen I., Azadegan A., Davis D.F. (2023). Resource Scarcity and Humanitarian Social Innovation: Observations from Hunger Relief in the Context of the COVID-19 Pandemic. J. Bus. Ethics.

[93] Baker P., Morgan A. (2012). DEFRA Project FO0108: Resilience of the Food Supply to Port Disruption.

[94] Revoredo-Giha C., Costa-Font M. (2022). The UK’s Fresh Produce Supply under COVID-19 and a No-Deal Brexit. LSE Business Review. https://blogs.lse.ac.uk/businessreview/2020/06/22/the-uks-fresh-produce-supply-under-covid-19-and-a-no-deal-brexit/.

[95] Ivanov D., Dolgui A. (2021). OR-methods for coping with the ripple effect in supply chains during COVID-19 pandemic: Managerial insights and research implications. Int. J. Prod. Econ..

[96] Shanks S., Van Schalkwyk M.C.I., McKee M. (2020). COVID-19 exposes the UK’s broken food system. BMJ.

[97] Hirvonen K., De Brauw A., Abate G.T. (2021). Food Consumption and Food Security during the COVID-19 Pandemic in Addis Ababa. Am. J. Agric. Econ..

[98] EIT Food (2020). COVID-19 Impact on Consumer Food Behaviours in Europe. https://www.eitfood.eu/media/news-pdf/COVID-19_Study_-_European_Food_Behaviours_-_Report.pdf.

[99] Clapp J., Moseley W.G. (2020). This food crisis is different: COVID-19 and the fragility of the neoliberal food security order. J. Peasant. Stud..

[100] Food Standards Agency (FSA) (2021). Food in a Pandemic. https://www.food.gov.uk/research/research-projects/food-in-a-pandemic.

[101] Purdam K., Garratt E.A., Esmail A. (2016). Hungry? Food Insecurity, Social Stigma and Embarrassment in the UK. Sociology.

[102] Lambie-Mumford H., Loopstra R., Lambie-Mumford H., Silvasti T. (2020). Food banks and the UK welfare state. The Rise of Food Charity in Europe.

[103] Garthwaite K.A., Collins P.J., Bambra C. (2015). Food for thought: An ethnographic study of negotiating ill health and food insecurity in a UK foodbank. Soc. Sci. Med..

[104] Caraher M., Cavicchi A. (2014). Old crises on new plates or old plates for a new crises? Food banks and food insecurity. Br. Food J..

[105] UK Government (2020). COVID-19 and Food Supply. Environment, Food and Rural Affairs Committee—House of Commons—Government Response. https://publications.parliament.uk/pa/cm5801/cmselect/cmenvfru/263/26306.htm.

[106] Altendorfer-Kaiser S. (2017). The Influence of Big Data on Production and Logistics: A Theoretical Discussion. Advances in Production Management Systems. The Path to Intelligent, Collaborative and Sustainable Manufacturing, Proceedings of the IFIP WG 5.7 International Conference, APMS 2017, Hamburg, Germany, 3–7 September 2017.

[107] Manning L., Soon J.M. (2016). Building strategic resilience in the food supply chain. Br. Food J..

[108] Hallegatte S. (2014). Economic Resilience Definition and Measurement. https://openknowledge.worldbank.org/handle/10986/18341.

[109] Hosseini S., Ivanov D., Dolgui A. (2019). Review of quantitative methods for supply chain resilience analysis. Transp. Res. Part E Logist. Transp. Rev..

[110] O’Meara L., Turner C., Coitinho D.C., Oenema S. (2022). Consumer experiences of food environments during the COVID-19 pandemic: Global insights from a rapid online survey of individuals from 119 countries. Glob. Food Secur..

[111] De Steenhuijsen Piters B., Termeer E., Bakker D., Fonteijn H., Brouweret H., Ribeiro-Barros A.I., Tevera D.L.F., Goulao L.F., Tivana L.D. (2021). Perspective Chapter: Food System Resilience—Towards a Joint Understanding and Implications for Policy. Food Systems Resilience.

[112] Shadbolt N., Olubode-Awosola F., Rutsito B. (2017). Resilience in dairy farm businesses; to ‘bounce without breaking’. J. Adv. Agric..

[113] Shadbolt N.M., Olubode-Awosola F. (2016). Resilience, Risk and Entrepreneurship. IFAMR.

[114] Shadbolt N., Olubode-Awosola F., Rutsito B. (2013). Resilience to ‘bounce without breaking’ in New Zealand dairy farm businesses. IFMA 19 Transform. Agric..

[115] Mu W., van Asselt E.D., Van der Fels-Klerx H.J. (2021). Towards a resilient food supply chain in the context of food safety. Food Control.

[116] Karienye D., Macharia J., Oguge N., Ayal D., Adeleke L., da Silva I. (2021). Adaptive capacity to mitigate climate variability and food insecurity of rural communities along River Tana Basin, Kenya. African Handbook of Climate Change Adaptation.

[117] Kogo B.K., Kumar L., Koech R. (2021). Climate change and variability in Kenya: A review of impacts on agriculture and food security. Environ. Dev. Sustain..

[118] Hamza I.A., Iyela A. (2012). Land use pattern, climate change, and its implication for food security in Ethiopia: A review. Ethiop. J. Environ. Stud. Manag..

[119] Folke C., Carpenter S.R., Walker B., Scheffer M., Chapin T., Rockström J. (2010). Resilience thinking: Integrating resilience, adaptability and transformability. Ecol. Soc..

[120] Janssen M., Chang B.P.I., Hristov H., Pravst I., Profeta A., Millard J. (2021). Changes in Food Consumption During the COVID-19 Pandemic: Analysis of Consumer Survey Data From the First Lockdown Period in Denmark, Germany, and Slovenia. Front. Nutr..

[121] Chiwona-Karltun L., Amuakwa-Mensah F., Wamala-Larsson C., Amuakwa-Mensah S., Abu Hatab A., Made N., Taremwa N.K., Melyoki L., Rutashobya L.K., Madonsela T. (2021). COVID-19: From health crises to food security anxiety and policy implications. Ambio.

[122] Fenner R., Cernev T. (2021). The implications of the COVID-19 pandemic for delivering the Sustainable Development Goals. Futures.

[123] Kowalska A., Lingham S., Maye D., Manning L., Bukalska E., Kijek T., Sergi D.S. (2023). Food security through the COVID-19 crisis and beyond—Poland: A case study. Modeling Economic Growth in Contemporary Poland.

